# Assisted suicide is a critical problem in psychiatry

**DOI:** 10.1192/j.eurpsy.2023.1859

**Published:** 2023-07-19

**Authors:** P. Liubov

**Affiliations:** Department of gerontopsychiatry, Moscow Research Institute of Psychiatry - branch of the V. Serbsky National Medical Research Center for Psychiatry and Narcology, Moscow, Russia, Moscow, Russian Federation

## Abstract

**Introduction:**

Active euthanasia is currently permitted in Netherlands, Belgium, Colombia, Luxembourg, Canada, Australia and India. Assisted suicide is allowed in Switzerland, Germany, South Korea, Japan, as well as in the states of Washington, Oregon, Colorado, Hawaii, Vermont, Montana, California of the USA.

The right to die is considered to be a basic human right. In 2018, 2,357 euthanasia procedures were carried out in Belgium, most of them for the elderly. Factors that contribute to the decision to euthanize older people are: existential crisis; loss of autonomy, dignity and control; worry about future loss of autonomy, dignity, and control; lack of understanding of the processes of dying; concerns about medical intervention and treatment at the end of life; increasing disunity between generations; decline of people’s spiritual culture and religious faith. In the Netherlands, euthanasia is allowed from the age of 12, sometimes for infants up to two years old. Some countries allow assisted suicide of the mentally ill.

The possibility of suicide for people «who are tired of life» is discussed.

**Objectives:**

Investigation of the ethical aspects of the role of a psychiatrist in the commission of assisted suicide

**Methods:**

In order to study ethical aspects of the role of a psychiatrist in assisted suicide, the materials of the Department of bioethics of UNESCO and the positions of the legislation of a number of countries were studied.

**Results:**

The decision about euthanasia is taken by a commission of 3 doctors, one of whom is a psychiatrist.

According to psychiatrists, the desire to die in a patient with a mental disorder should be considered the same as the desire of a patient with cancer.

The role of psychiatrists in euthanasia and assisted suicide is reversed. These are: the study of assisted suicides, assistance in their implementation and popularization, determination of legal capacity to permit suicide, creation of a psychotherapeutic space and providing psychological assistance to patients in the process of dying, writing of a prescription for a lethal drug, consulting patients and their families.

**Conclusions:**

The role of psychiatrists in Russia is to prevent suicides and treat patients with suicidal tendencies. Assistance by a psychiatrist in the suicide is a critical problem in psychiatry.

**Disclosure of Interest:**

None Declared

